# Knockdown of Long Non-coding RNA SNGH3 by CRISPR-dCas9 Inhibits the Progression of Bladder Cancer

**DOI:** 10.3389/fmolb.2021.657145

**Published:** 2021-03-29

**Authors:** Yu Cao, Qiong Hu, Ruiming Zhang, Ling Li, Mingjuan Guo, Huiling Wei, Li Zhang, Jianfeng Wang, Chunjing Li

**Affiliations:** ^1^Ningxiang Hospital Affiliated to Hunan University of Traditional Chinese Medicine, Ningxiang, China; ^2^Medical Basic Teaching Experiment Center, College of traditional Chinese Medicine, Hunan University of Chinese Medicine, Changsha, China; ^3^Department of Urology, Affiliated Foshan Maternal and Child Healthcare Hospital, Southern Medical University, Foshan, China

**Keywords:** SNGH3, bladder cancer, CRISPR-dCas9, lncRNA, therapeutic target

## Abstract

Recent research evidence documents that lncRNAs (long non-coding RNAs lncRNAs) play a pivotal role in the tumorigenesis and development of tumors. LncRNA SNGH3 (small nucleolar RNA host gene 3) is highly expressed in numerous forms of cancer, serving as an oncogene in cancer progression. Nonetheless, the clinical relationship, along with the mechanism of SNGH3 in bladder cancer, have not been studied. Herein, the findings exhibited upregulation of SNGH3 in bladder cancer tissues, along with the cell lines. Furthermore, overexpressed SNGH3 was positively linked to the TNM stage, as well as the histological grade of bladder cancer. Moreover, the silencing of SNGH3, using CRISPR-dCas9, suppressed cell growth along with migration, but elevated bladder cancer cell apoptosis. In summary, we established that SNGH3 serves as a bladder cancer oncogene and could be employed as a prospective diagnostic marker for clinical use, and is also a therapeutic target for CRISPR-mediated gene therapy.

## Introduction

Human bladder cancer, which has high incidence and a high mortality, is the most frequent type of the urinary system tumors. According to statistics, there are approximately 549,000 new cases of bladder cancer and 200,000 people died of the disease in 2018 (Bray et al., 2018). Although significant advancements have been made in the treatment of bladder cancer in recent years, the 5 year survival rate and prognosis of patients with advanced bladder cancer remain low (Kamat et al., 2016; Dy et al., 2017; Massari et al., 2018). Early diagnosis and treatment of bladder cancer is strongly linked to the prognosis of patients. Frequent relapse along with distant metastasis of bladder cancer are the primary causes of treatment failure. However, the exact molecular biology of bladder cancer remains unknown. Therefore, it is critical to find new effective biological targets for the diagnosis, as well as the treatment of bladder cancer.

Long non-coding RNA (lncRNA) is a non-coding RNA molecule with a length of more than 200 nt. Because lncRNAs lack (ORFs) open reading frames, they cannot be translated into proteins (Ponting et al., 2009). Recent research findings have documented that lncRNAs have a core role in cell proliferation, apoptosis, infiltration, and other biological processes, as well as gene regulation, chromatin remodeling, and other molecular levels (Tsai et al., 2010; Quinodoz and Guttman, 2014). In addition, a large number of lncRNAs participate in tumor development along with metastasis (Huarte, 2015). The expression characteristics of lncRNAs have obvious tissue or cell specificity. They can be used as oncogenic factors or tumor repressors (Evans et al., 2016; Chen Y. et al., 2017). In many classical instances, lncRNAs are employed as guides or molecular scaffolds that modulate protein-protein or DNA-protein cross talks, as enhancers that modulate transcription, or as miRNA (microRNA) sponges to adsorb miRNAs (Jandura and Krause, 2017; Marchese et al., 2017). lncRNA can also be used as readouts of active cellular genetic programs or conductors of distinct cellular signaling events, can assess the pathological state of cancers, and offers prognostic ability for cancer patients (Engreitz et al., 2016). For example, lncRNA MALAT-1 has been found to be upregulated in various malignant tumors, and upregulated MALAT-1 is remarkably linked to worse OS (overall survival) or DFS (disease-free survival) in individuals with gastrointestinal carcinomas (Chen D. et al., 2017; Wu et al., 2018). High expression of lncRNA HOTAIR in primary, as well as metastatic breast cancer indicates dismal prognosis. It promotes the infiltration along with the metastasis of breast cancer cells by mediating PRC2 relocation and modulating H3K27 methylation (Milevskiy et al., 2016; Portoso et al., 2017). LncRNA-HOXD-AS1 functions as a sponge of miRNA and competitively bind with miR-130a, thereby upregulating the expression of the EF28 transcription factor, and enhancing the migration and infiltration of glioma cells (Chen et al., 2018). Therefore, it is extremely necessary to conduct a comprehensive genomic and cellular functional study on the changes of lncRNA in tumor development along with metastasis, which will help to uncover new cancer diagnosis and treatment targets.

The dysregulation of lncRNAs is closely linked to multiple cancer types, such as small nucleolar RNA host gene (SNHG) related lncRNA. Recently, accumulating evidence suggests that small nucleolar RNA host genes of lncRNAs participate in the onset of cancer, accelerating the progression of cancer cells. For instance, lncRNA SNHG1 promotes the growth of colon and lung cancer by enhancing the transcriptional level of neighboring gene SLC3A2 and accelerating the phosphorylation of the PI3K/Akt pathway (Sun et al., 2017). The oncogenic lncRNA, SNHG6 enhances genomic hypomethylation by inhibiting the generation of SAMe. Moreover, SNHG6 sponges miR-1297 to increase MAT2A expression, upregulating MAT2A expression in hepatocellular carcinoma (Guo et al., 2018). Shan et al. (2018) documented that lncRNA SNHG7 was highly expressed in colorectal cancer and increased up regulated SNHG7 expression, promoted the cell growth and migration by acting as the miR-216b sponge to affect the expression and GALNT1 and EMT markers.

To the best of our knowledge, there is no study showing the biological function of lncRNA SNHG3 in bladder cancer. Herein, the data revealed lncRNA SNHG3 upregulation in bladder cancer tissues, as well as cell lines in contrast with the matched bladder non-malignant tissues and SV-HUC1 cells, respectively. Elevated SNHG3 expression was positively linked to the TNM stage along with the histological grade of individuals with bladder cancer. In addition, upregulated SNHG3 expression was shown to result in the dismal prognosis of bladder cancer patients, causing lower OS and DFS. Further functional assays illustrated that SNHG3 silencing, by CRISPR-dCas9, repressed the bladder cancer cell growth, as well as migration and enhanced cell apoptosis.

## Materials and Methods

### Patient Samples

Overall, 41 individuals who were diagnosed with bladder cancer and who had undergone partial or radical cystectomy at the Affiliated Foshan Maternal and Child Healthcare Hospital were enrolled in the study. All the bladder cancer tissues along with the matching neighboring non-malignant tissues were stored in RNA later and then immediately snap-frozen in liquid nitrogen.

All subjects who enrolled in the present study approved this research and provided written informed consent. In addition, we performed the study with the approval of the Research Ethics Committee of the Affiliated Foshan Maternal and Child Healthcare Hospital.

### Cell Culture

The 5637, SW780, and T24 human bladder cancer cells along with the SV-HUC-1 human non-malignant bladder epithelial cells were all supplied by ATCC. The RPMI-1640 Medium (Gibco) augmented with 10% FBS (Invitrogen, Carlsbad, CA, United States) was utilized to grow all the cells under 37°C and 5% CO_2_ growth conditions.

### Cell Transfection

SW780 along with the 5637 cells were transiently inserted with specific sgRNA targeting on lncRNA SNHG3 through transfection. The SNHG3 sgRNA sequence was listed as follows: sgRNA-1: 5′-GGACTTCCGGGCACTTCGTA-3′; sg-RNA-2: 5′-GGACTTCCGGGCACTTCGTA-3′; sgRNA-3: 5′-GATGCTTGCCACCGGAGTTG-3′. SNHG3 sgRNA (sg-SNHG3), and the sg-NC (negative control) were synthesized by GenePharma (Suzhou, China). The Lipofectamine 3000 (Invitrogen, Carlsbad, CA) was employed to transfect the bladder cancer cells as described by the manufacturer. Transfection was done after the cultured cells attained 50–70% confluence in six-well plates. We harvested the transfected cells for real-time quantitative PCR after 48 h.

### RT-qPCR

The Trizol reagent (Invitrogen, Carlsbad, CA, United States) was utilized to isolate total RNA from the bladder cancer tissues and cells as described in the manual provided by the manufacturer. After that, cDNA was processed from 1 μg total RNA with the PrimeScript RT Reagent Kit with gDNA Eraser (Takara, Dalian, China) *via* reverse transcription. Subsequently, 2 μl of cDNA was employed as the template in qPCR process consisting of 10 μl of SYBR Green PCR kit mix (Takara, Dalian, China), 0.5 μl specific forward primer, 0.5 μl specific reverse primer, and 7 μl of deionized water. This assay was carried out and analyzed on the ABI7000 system (Applied Biosystems, Foster City, CA, United States). The GAPDH gene served as the internal standard. Each sample was assayed in triplicate.

### Cell Proliferation Assessment

The CCK along with the MTT assays were employed to study cell proliferation. At 48 h after transfection with sg-SNHG3 or sg-NC, we harvested the cells and then inoculated them in a 96-well plate for 24 h. Afterward, 10 μl CCK-8 reagent was introduced to every well at intervals of 0, 24, 48, and 72 h post cell attachment. The cells were cultured in darkness for 1 h. Next, a micro-plate reader (Bio-Rad, Hercules, CA, United States) was employed to determine the OD values at 450 nm. The MTT assay was employed to validate the CCK-8 assay data. In brief, we introduced 10 μl of MTT reagent to every well at 0, 24, 48, and 72 h post cell attachment. The cells were cultured in darkness for 1 h and then 100 μl of DMSO was introduced. A micro-plate reader (Bio-Rad, Hercules, CA, United States) was employed to determine the OD values at 450 nm after 10 min of vibration.

### Cell Migration Assessment

Cell migration was explored by the scratch assay and transwell assay. The bladder cancer cells were inserted with specified sgRNA oligonucleotides when cells attained 90% confluence. At 24 h after transfection, we used a sterile 200 μl pipette tip to generate a clear line in the wells. The images of mobilized cells were captured from every well with a digital camera at 0 and 24 h post scratching for bladder cancer cells. Relative migration distance was measured using Photoshop software. To validate the scratch assay data, a transwell assay was also conducted. Approximately 10,000 transfected cells enriched with 100 μl serum-free medium were planted into the upper compartments (24-well plate, pore size 8 μm, Corning). 500 μl of DMEM medium enriched with 10% FBS was introduced into the lower compartments in each well. The cells were grown for 24 h and then the chambers were rinsed with PBS twice. Afterward, 4% paraformaldehyde fixation of the cells under the lower compartment surface was done for 30 min. Subsequently, 0.1% crystal violet staining of the cells was done for 40 min, followed by rinsing twice with PBS. An inverted microscope was employed to observe the migrated cells, which were imaged. Lastly, 600 μl of 33% glacial acetic acid was introduced to each chamber to wash out the crystal violet and was then incubated for 30 min. 100 μl of scrubbing solution was added to 96-well plates and a microplate reader was employed to examine the absorbance at 570 nm.

### Caspase 3 ELISA Assay

Caspase 3 ELISA analysis was employed to investigate cell apoptosis. 5637 and SW780 bladder cancer cells were inserted with sg-SNHG3 or sg-NC in 12-well plates through transfection. Forty-eight hours post transfection, the activity of capase-3 was employed to assay the apoptosis caused by decreased SNHG3 expression as described in the manufacturer manual (Cusabio, Wuhan, China). Absorbance was examined by a microplate reader. The ratios between the OD values of the sg-SNHG3 cell transfects and those of the sg-NC cell transfects were employed to present the results. Experiments were replicated at least three times.

### Statistical Analyses

All these statistical analyses were executed with SPSS 20.0. The Paired samples *t*-test was employed to explore the SNHG3 RNA expression differences between bladder cancer tissues and matching non-malignant tissues. Moreover, the differences in SNHG3 RNA content between cancer cells and SV-HUC1 were assessed using the independent samples *t*-test. The results of the CCK-8 assay and MTT assay were explored by the independent samples *t*-test at each time point. Cell migration along with an apoptosis assay were assessed by the independent samples *t*-test. *P* < 0.05 designated statistical significance.

## Results

### LncRNA SNHG3 Is Highly Expressed in Bladder Cancer Tissues and Cell Lines

**FIGURE 1:**
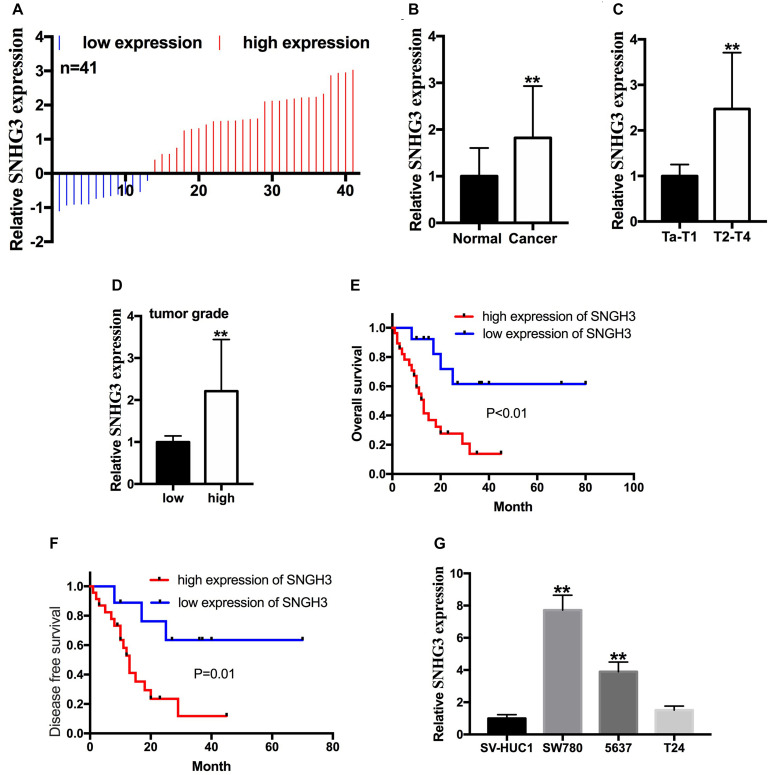
SNGH3 was highly expressed in bladder cancer tissues, and cell lines. **(A)** 41 bladder cancer samples were analyzed herein. SNHG3 was highly expressed in 28 of 41 bladder cancer tissue samples. **(B)** Total SNHG3 expression in bladder cancer tissue was remarkably higher in contrast with that in matched neighboring non-malignant tissues. **(C)** SNGH3 RNA content was higher in T2-T4 stage cancers in contrast with those in Ta-T1 cancers. **(D)** SNGH3 RNA content was higher in high grade cancers in contrast with those in low grade cancers. **(E)** The association of SNGH3 content with OS. **(F)** The association of SNGH3 RNA content with DFS. **(G)** SNGH3 was overexpressed in SW780 and 5637 cells in contrast with that in SV-HUC1. Data were indicated as means ± SD from three independent experiments (**P < 0.01).

The relative lncRNA SNHG3 expression was determined by performing a qRT-PCR assay in bladder cancer tissues, as well as the cells. In contrast with the matching non-malignant bladder peritumoral tissues, SNHG3 expression was highly expressed in 68.3% (28 of 41) of bladder cancer tissues (Figure 1A). The SNHG3 expression fold change (bladder cancer tissues/corresponding non-malignant tissues) in total bladder cancer patients is shown in Figure 1B. Moreover, elevated SNHG3 content was strongly linked to the TNM stage along with histological grade of individuals with bladder cancer, but irrelevant to other clinical features, including gender, lymph node metastasis, age, distant metastasis, as well as tumor size (Table 1). The expression differences based on the TNM stage and histological grade were shown as follows. The SNHG3 expression fold change in T2-T4 stage bladder cancer tissues was 2.47 times that in Ta-T1 stage bladder cancer tissues (Figure 1C). The SNHG3 expression fold change in high grade bladder cancer tissues was 2.21 times that in low grade bladder cancer tissues (Figure 1D). Moreover, the OS (Figure 1E) and DFS (Figure 1F) in the SNHG3 overexpression group was remarkably lower in contrast to that in the SNHG3 down-regulation group, indicating that SNHG3 could be applied as a prognostic indicator for bladder cancer. Lastly, we established that SNHG3 was highly expressed in the bladder cancer cells (5637 and SW780) in contrast to that in SV-HUC1 (Figure 1G), implying that SNHG3 is a bladder cancer oncogene. Hence, 5637 and SW78O cells were selected to perform functional experiments.

**TABLE 1:** Relationship of SNGH3 expression with clinicopathological features of bladder cancer patients.

Parameters	Group	Total	Expression		*P*-value
			High	Low	
Gender	Male	26	16	10	0.221
	Female	15	12	3	
Age (years)	<60	10	6	4	0.779
	≫60	31	22	9	
Tumor size	<3	14	10	4	0.756
	≫3	27	18	9	
TNM stage	Ta + Tis + TI	12	5	7	0.009
	T2 or above	29	24	5	
Grade	Low	8	2	6	0.003
	High	33	26	7	
Lymph node metastasis	No	24	15	9	0.344
	N1 or above	17	13	4	
Distant metastasis	No	38	25	13	0.22
	Yes	3	3	0	

**P < 0.05 signified statistical significance.**

### Specific sgRNA Inhibited the Expression of SNHG3

**FIGURE 2:**
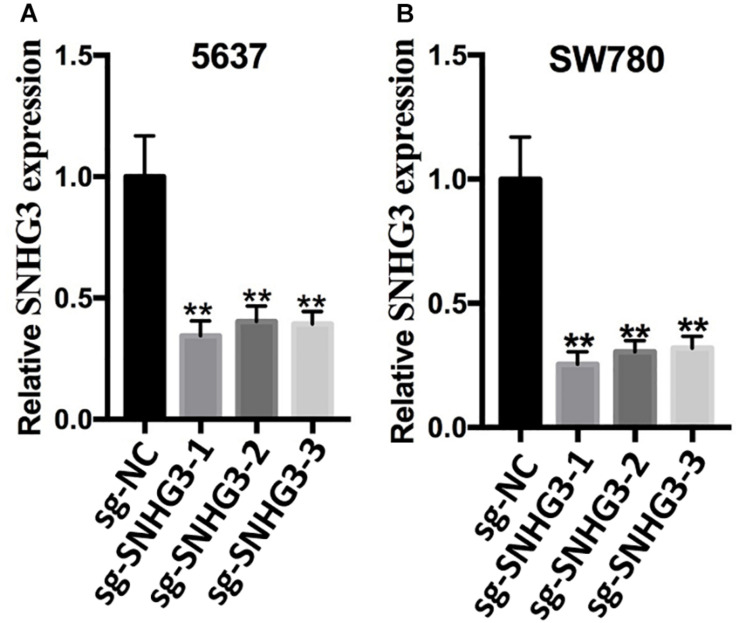
Knockdown of SNGH3 expression. **(A)** Silencing of SNGH3 with sgRNA in 5637 cell. **(B)** Silencing of SNGH3 with sgRNA in SW780 cell. Data were indicated as means ± SD from three independent experiments (** denotes *P* < 0.01). sg: sgRNA.

According to the promoter sequence of SNHG3, we designed three different sgRNAs (sg-SNHG3-3, sg-SNHG3-2, as well as sg-SNHG3-1) targeting SNHG3 and evaluated their efficiencies in 5637 and SW780 bladder cancer cells inserted with a sg-SNHG3 or sgRNA negative control (sg-NC). At 48 h post transfection, SNHG3 expression was measured. As shown in Figures 2A,B, sg-SNHG3-1 induced the maximal knockdown effect of SNHG3 in 5637 and SW780 cells. Therefore, sg-SNHG3-1 was used to perform the functional experiments.

### Suppression of SNHG3 Repressed Cell Proliferation

**FIGURE 3:**
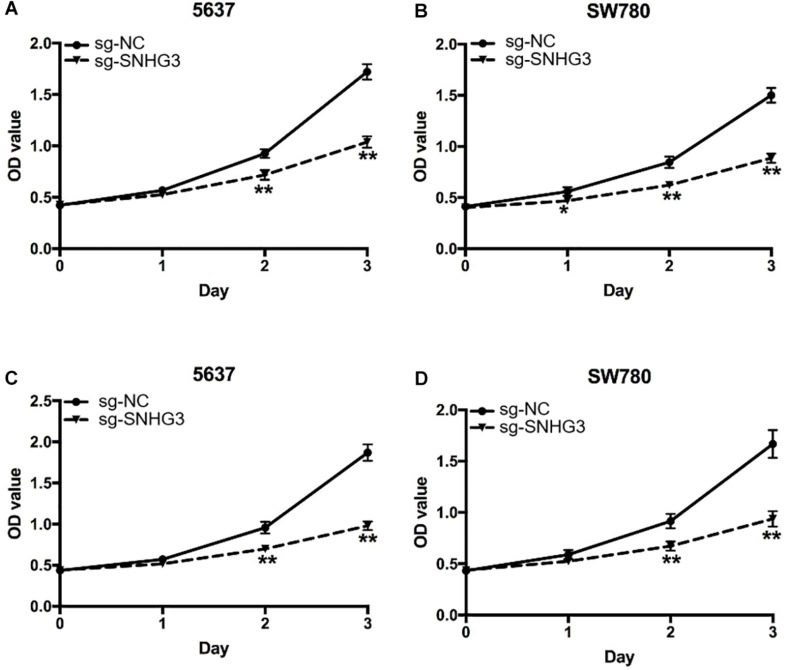
SNGH3 silencing repressed *in vitro* cell proliferation. **(A,B)** CCK-8 assay illustrated that repression of SNGH3 reduced the growth of 5637 and SW780 cells. **(C,D)** MTT assay illustrated that SNGH3 silencing dramatically repressed the growth of 5637 and SW780 cells. Data were indicated as means ± SD from three independent experiments (** denotes *P* < 0.01, * denotes *P* < 0.05). sg: sgRNA.

To assess the influence of SNHG3 on cell growth, CCK-8 assay along with MTT assay were carried out. As indicated in Figures 3A,B, the results of CCK-8 demonstrated that sg-SNHG3 repressed cell growth remarkably in both 5637 and SW780 cells. In addition, the results of MTT were congruent with the results of the CCK-8 assay data. As indicated in Figures 3C,D, SNHG3 silencing remarkably reduced the growth curve of SW780 and 5637 cells. Collectively, the data confirmed that suppression of SNHG3 inhibited bladder cancer cell growth.

### SNHG3 Silencing Promoted Cell Apoptosis

**FIGURE 4:**
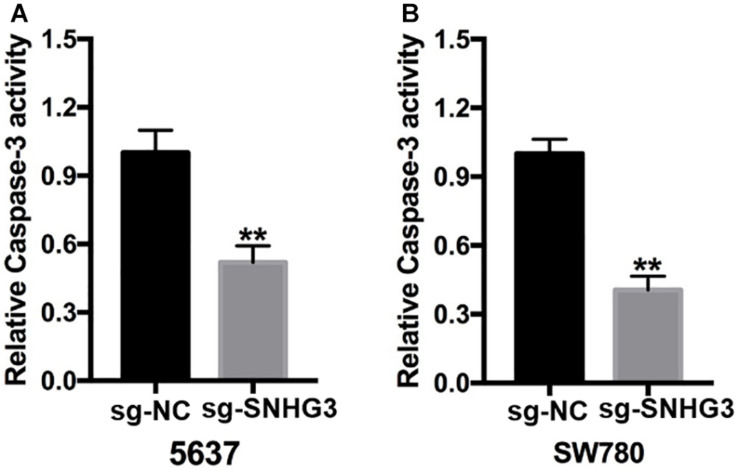
SNGH3 silencing triggered cell apoptosis. **(A)** Relative caspase activity in the 5637 cells transfected with sg-SNGH3 was remarkably increased in contrast with that in the negative control group. **(B)** Relative caspase-3 activity in the sg-SNGH3 transfected SW780 cells was remarkably up-regulated in contrast with that in the negative control group. Data were indicated as means ± SD from three independent experiments (**P < 0.01). sg: sgRNA.

Forty-eight hours post transfection, we harvested the bladder cancer cells and then used them to perform the Capase-3 ELISA assay. As indicated in Figures 4A,B, there were remarkably elevated relative Capase-3 activity in sg-SNHG3 bladder cancer cells in contrast with sg-NC cell transfects.

### Knockdown of SNHG3 Inhibited Cell Migration and Infiltration

**FIGURE 5:**
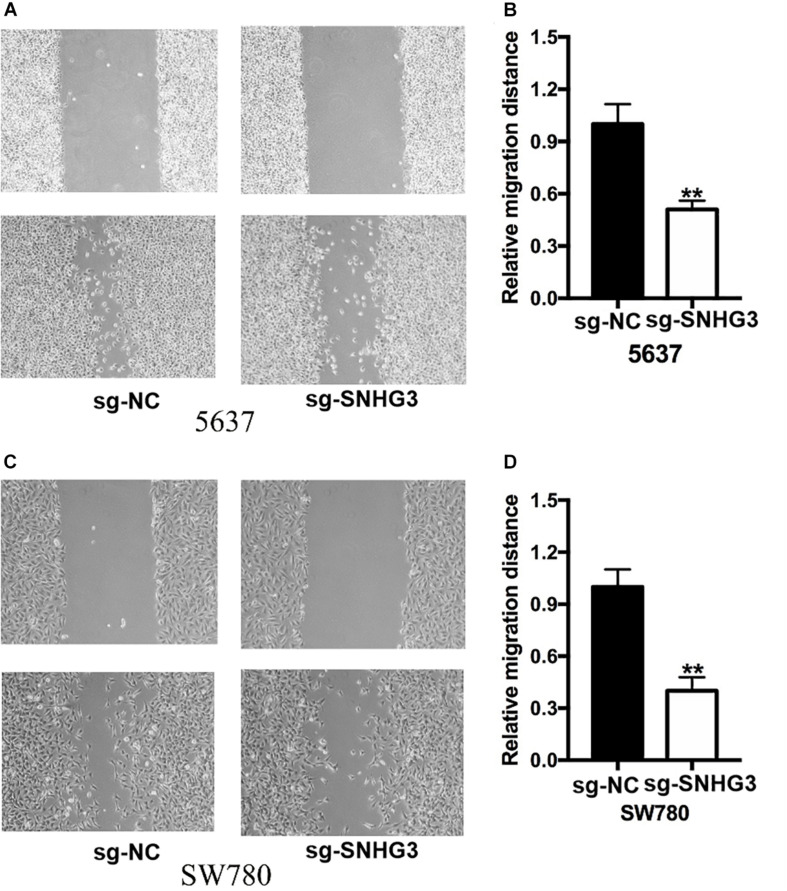
Silencing of SNGH3 repressed migration of cells. The wound healing experiment was applied to examine bladder cancer cell migration. **(A)** Suppression of SNGH3 caused a slower closing of scratch wounds in 5637 cells. **(B)** Reduced cell migration was reported in 5637 cells treated with sg-SNGH3. **(C)** Knockdown of SNGH3 caused a slower closing of scratch wounds in SW780 cells. **(D)** Cell migration inhibition was assayed in SW780 cells treated with sg-SNGH3. Data were indicated as means ± SD from three independent experiments (** denotes *P* < 0.01). sg: sgRNA.

**FIGURE 6:**
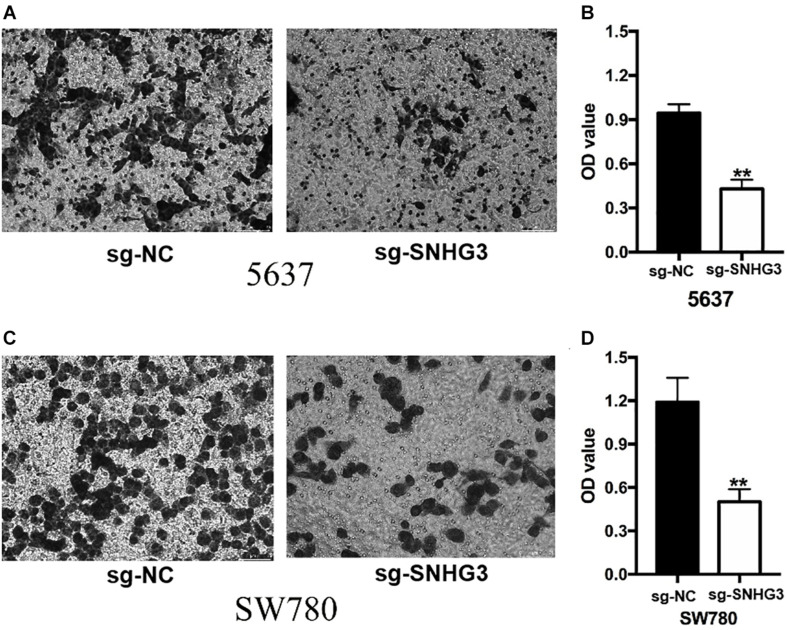
SNGH3silencing inhibited cell infiltration. Transwell assay was employed to explore bladder cancer cell infiltration. **(A)** Images illustrating transwell assay in 5637 cells. **(B)** Inhibition of SNGH3 reduced the invasion of 5637 cells. **(C)** Images illustrating wound healing assay in SW780 cells. **(D)** Inhibition of SNGH3 reduced the invasion of 5637 cells. Data were indicated as means ± SD from three independent experiments (** denotes *P* < 0.01). sg: sgRNA.

The scratch assay along with the transwell assay were employed to verify the effect of SNHG3 on cell migration, as well as infiltration. The scratch experiment showed that bladder cancer cells transfected with sg-SNHG3 exhibited a slower rate of closing of scratched wounds in contrast with the sg-NC groups (Figures 5A,B). Further data analysis indicated that the relative migration distance in the sg-SNHG3 group was decreased by 49% in the 5637 cell (Figure 5C) and reduced by 60% in the SW780 cell (Figure 5D). The Transwell assay was additionally conducted to explore bladder cancer cell invasion. In contrast with the sg-NC group, cell infiltration potential of the sg-SNHG3 group was extremely inhibited in the 5637 (Figure 6A) and SW780 cells (Figure 6B). The OD value of washing-up liquid in the sg-SNHG3 transfected 5637 (Figure 6C) and SW780 cells (Figure 6D) was remarkably lower in contrast with the sg-NC group. Altogether, these data revealed that SNHG3 silencing repressed migration along with the infiltration of bladder cancer cells.

## Discussion

LncRNAs are a kind of non-protein coding RNAs with a length of about 200 nucleotides. Mounting research evidence suggest that lncRNAs are involved with gene expression regulation by affecting the gene transcription, post-transcriptional process, and chromatin modification (Meller et al., 2015; Engreitz et al., 2016). Furthermore, lncRNAs can act as competitive endogenous RNA to capture microRNA which can suppress the transcriptional and translational process by docking to the 3′UTR region of mRNA (Thomson and Dinger, 2016). Hence, lncRNAs participate in modulatory roles in nearly all the biological processes, constituting cell growth, tissue, as well as organ development, cell apoptosis, energy metabolism, and so on (Kopp and Mendell, 2018; Krause, 2018). However, dysregulation of lncRNAs will cause the disorder of the internal environment and multiple types of human diseases, especially cancers. Aberrant expression of lncRNAs can enhance the growth, infiltration, and drug resistance of cancer cells. Moreover, the disorder of lncRNAs can disturb the cell apoptosis process (Parasramka et al., 2016; Schmitt and Chang, 2016). For instance, upregulation of lncRNA HOXD-AS1 has been documented in bladder cancer tissues, as well as cells, and HOXD-AS1 can enhance the cell growth and migration, but can repress cell apoptosis during the progression of bladder cancer (Li et al., 2016). LncRNA SPRY4-IT1 enhances the growth along with the metastasis of bladder cancer cells though sponging miR-101-3p to increase EZH2 expression (Li et al., 2017; Liu et al., 2017). As a recently identified lncRNA, multiple previous studies were summarized to characterize the oncogenic features of lncRNA SNHG3, a 2,238 nt in length lncRNA located on chromosome 1p36.1. SNHG3 is overexpressed in hepatocellular carcinoma (HCC) tissues in contrast with the matched non-malignant tissues. SNHG3 RNA content is positively correlated with portal vein tumor thrombus tumor size and tumor relapse of HCC patients. Increased expression of SNHG3 causes lower OS, poorer recurrence-free survival, as well as lower DFS (Zhang et al., 2016). Zhang et al. (2018) demonstrated that overexpression of SNHG3 facilitates cell infiltration, the epithelial−mesenchymal transition (EMT) progress, along with sorafenib resistance by sponging miR−128 to up-regulate CD151 expression in HCC cells. Huang et al. (2017) extracted the data from the TCGA dataset and documented that SNHG3 was up-regulated in CRC tissues and cells in contrast with neighboring non-malignant tissues and normal cells, respectively. SNHG3 silencing repressed cell growth and tumor growth by capturing miR-182-5p, which can suppress c-Myc expression. Fei et al. (2018) found that SNHG3 could recruit EZH2 to the KLF2 and p21 promoter region to suppress KLF2 and p21 expression in the malignant progression of glioma cells. Li et al. (2018) also extracted the data of ovarian cancer from the TCGA data resource and documented that SNHG3 was closely linked to the OS and energy metabolism. Further analysis suggested that SNHG3 absorbed miRNAs and EIF4AIII to affect the expression of UQCRH, IDH2, PKM, and PDHB and in the energy metabolism related cascades. Moreover, SNGH3 was remarkably overexpressed in lung adenocarcinoma samples in contrast with neighboring samples. Forced SNGH3 expression facilitated A549 cell growth and inhibited A549 cell apoptosis (Liu et al., 2018). In general, SNGH3 is overexpressed in HCC, CRC, glioma, and lung adenocarcinoma. SNGH3 promotes the malignant phenotype of these cancer cells *via* sponging miRNA or acting as a protein scaffold.

CRISPR-dCas9 is a new gene regulation system which was developed in 2013 (Vigouroux et al., 2018; Lee et al., 2021). It was first used in bacteria and was later widely used in human cells. The mutated dCas9 protein cannot cut DNA but can bind to the promoter region or ORF region of the gene, thus hindering the normal transcription of the gene through steric hindrance. This tool is very suitable for studying the function of lncRNAs.

To the best of our knowledge, this is the first report to explore the role of lncRNA SNGH3 in bladder cancer. Herein, it was discovered that SNGH3 was highly expressed in bladder cancer tissues along with the cell lines, in contrast with the matched non-malignant tissue and cell. Elevated SNGH3 expression was directly linked to the TNM stage, as well as the histological grade of bladder cancer. Moreover, bladder cancer individuals with high SNGH3 expression showed worse OS and DFS. The differential SNGH3 expression trends between bladder cancer tissues and non-malignant tissues and the correlation of SNGH3 with clinic pathological data indicate that SNGH3 emerges as a pivotal player in the onset and progression of bladder cancer. A further functional assay showed that cell proliferation, repression reduced motility, and escalated apoptosis were remarkably detected in the sg-SNGH3 transfected group. These findings demonstrated that SNGH3 played oncogenic roles in the development of bladder cancer. Furthermore, SHGH3 may act as prospective prognostic biomarker and treatment target for bladder cancer. Targeting on SNGH3 using CRISPR technology may be a valuable strategy to fight against bladder cancer.

## Data Availability Statement

The original contributions presented in the study are included in the article/supplementary material, further inquiries can be directed to the corresponding author/s.

## Ethics Statement

The studies involving human participants were reviewed and approved by the Research Ethics Committee of the Affiliated Foshan Maternal and Child Healthcare Hospital. The patients/participants provided their written informed consent to participate in this study.

## Author Contributions

YC, QH, RZ, LL, MG, HW, LZ, and JW performed the biological experiments and data analyses. CL designed and supervised the project. All authors contributed to the article and approved the submitted version.

## Conflict of Interest

The authors declare that the research was conducted in the absence of any commercial or financial relationships that could be construed as a potential conflict of interest.

## References

[B1] Bray F. Ferlay J. Soerjomataram I. Siegel R. L. Torre L. A. Jemal A. (2018). Global cancer statistics 2018: GLOBOCAN estimates of incidence and mortality worldwide for 36 cancers in 185 countries. *CA Cancer J. Clin.* 68 394–424. 10.3322/caac.21492 30207593

[B2] Chen D. Liu L. Wang K. Yu H. Wang Y. Liu J. (2017). The role of MALAT-1 in the invasion and metastasis of gastric cancer. *Scand. J. Gastroenterol.* 52 790–796. 10.1080/00365521.2017.1280531 28276823

[B3] Chen Y. Xie H. Zou Y. Lai X. Ma L. Liu Y. (2017). Tetracycline-controllable artificial microRNA-HOTAIR + EZH2 suppressed the progression of bladder cancer cells. *Mol. Biosyst.* 13 1597–1607. 10.1039/c7mb00202e 28671703

[B4] Chen Y. Zhao F. Cui D. Jiang R. Chen J. Huang Q. (2018). HOXD-AS1/miR-130a sponge regulates glioma development by targeting E2F8. *Int. J. Cancer* 142 2313–2322. 10.1002/ijc.31262 29341117

[B5] Dy G. W. Gore J. L. Forouzanfar M. H. Naghavi M. Fitzmaurice C. (2017). Global Burden of Urologic Cancers, 1990-2013. *Eur. Urol.* 71 437–446. 10.1016/j.eururo.2016.10.008 28029399

[B6] Engreitz J. M. Ollikainen N. Guttman M. (2016). Long non-coding RNAs: spatial amplifiers that control nuclear structure and gene expression. *Nat. Rev. Mol. Cell Biol.* 17 756–770. 10.1038/nrm.2016.126 27780979

[B7] Evans J. R. Feng F. Y. Chinnaiyan A. M. (2016). The bright side of dark matter: lncRNAs in cancer. *J. Clin. Invest.* 126 2775–2782. 10.1172/jci84421 27479746 PMC4966302

[B8] Fei F. He Y. He S. He Z. Wang Y. Wu G. (2018). LncRNA SNHG3 enhances the malignant progress of glioma through silencing KLF2 and p21. *Biosci. Rep.* 38:BSR20180420. 10.1042/BSR20180420 PMC6127675 30042166

[B9] Guo T. Wang H. Liu P. Xiao Y. Wu P. Wang Y. (2018). SNHG6 Acts as a Genome-Wide Hypomethylation Trigger via Coupling of miR-1297-Mediated S-Adenosylmethionine-Dependent Positive Feedback Loops. *Cancer Res.* 78 3849–3864. 10.1158/0008-5472.can-17-3833 29844127

[B10] Huang W. Tian Y. Dong S. Cha Y. Li J. Guo X. (2017). The long non-coding RNA SNHG3 functions as a competing endogenous RNA to promote malignant development of colorectal cancer. *Oncol. Rep.* 38 1402–1410. 10.3892/or.2017.5837 28731158 PMC5549033

[B11] Huarte M. (2015). The emerging role of lncRNAs in cancer. *Nat. Med.* 21 1253–1261. 10.1038/nm.3981 26540387

[B12] Jandura A. Krause H. M. (2017). The New RNA World: Growing Evidence for Long Noncoding RNA Functionality. *Trends Genet.* 33 665–676. 10.1016/j.tig.2017.08.002 28870653

[B13] Kamat A. M. Hahn N. M. Efstathiou J. A. Lerner S. P. Malmstrom P. U. Choi W. (2016). Bladder cancer. *Lancet* 388 2796–2810. 27345655 10.1016/S0140-6736(16)30512-8

[B14] Kopp F. Mendell J. T. (2018). Functional Classification and Experimental Dissection of Long Noncoding RNAs. *Cell* 172 393–407. 10.1016/j.cell.2018.01.011 29373828 PMC5978744

[B15] Krause H. M. (2018). New and Prospective Roles for lncRNAs in Organelle Formation and Function. *Trends Genet.* 34 736–745. 10.1016/j.tig.2018.06.005 30017312

[B16] Lee M. H. Lin C. C. Thomas J. L. Li J. A. Lin H. Y. (2021). Cellular reprogramming with multigene activation by the delivery of CRISPR/dCas9 ribonucleoproteins via magnetic peptide-imprinted chitosan nanoparticles. *Mater. Today Bio.* 9:100091. 10.1016/j.mtbio.2020.100091 33521619 PMC7820544

[B17] Li J. Chen Y. Chen Z. He A. Xie H. Zhang Q. (2017). SPRY4-IT1: A novel oncogenic long non-coding RNA in human cancers. *Tumour Biol.* 39:1010428317711406. 10.1177/1010428317711406 28651500

[B18] Li J. Zhuang C. Liu Y. Chen M. Chen Y. Chen Z. (2016). Synthetic tetracycline-controllable shRNA targeting long non-coding RNA HOXD-AS1 inhibits the progression of bladder cancer. *J. Exp. Clin. Cancer Res.* 35:99. 10.1186/s13046-016-0372-5 PMC4915162 27328915

[B19] Li N. Zhan X. Zhan X. (2018). The lncRNA SNHG3 regulates energy metabolism of ovarian cancer by an analysis of mitochondrial proteomes. *Gynecol. Oncol.* 150 343–354. 10.1016/j.ygyno.2018.06.013 29921511

[B20] Liu D. Li Y. Luo G. Xiao X. Tao D. Wu X. (2017). LncRNA SPRY4-IT1 sponges miR-101-3p to promote proliferation and metastasis of bladder cancer cells through up-regulating EZH2. *Cancer Lett.* 388 281–291. 10.1016/j.canlet.2016.12.005 27998761

[B21] Liu L. Ni J. He X. (2018). Upregulation of the Long Noncoding RNA SNHG3 Promotes Lung Adenocarcinoma Proliferation. *Dis. Markers* 2018:5736716. 10.1155/2018/5736716 PMC6081568 30154938

[B22] Marchese F. P. Raimondi I. Huarte M. (2017). The multidimensional mechanisms of long noncoding RNA function. *Genome Biol.* 18:206. 10.1186/s13059-017-1348-2 PMC5663108 29084573

[B23] Massari F. Santoni M. di Nunno V. Cheng L. Lopez-Beltran A. Cimadamore A. (2018). Adjuvant and neoadjuvant approaches for urothelial cancer: Updated indications and controversies. *Cancer Treat. Rev.* 68 80–85. 10.1016/j.ctrv.2018.06.002 29886353

[B24] Meller V. H. Joshi S. S. Deshpande N. (2015). Modulation of Chromatin by Noncoding RNA. *Annu. Rev. Genet.* 49 673–695. 10.1146/annurev-genet-112414-055205 26631517

[B25] Milevskiy M. J. Al-Ejeh F. Saunus J. M. Northwood K. S. Bailey P. J. Betts J. A. (2016). Long-range regulators of the lncRNA HOTAIR enhance its prognostic potential in breast cancer. *Hum. Mol. Genet.* 25 3269–3283. 27378691 10.1093/hmg/ddw177 PMC5179926

[B26] Parasramka M. A. Maji S. Matsuda A. Yan I. K. Patel T. (2016). Long non-coding RNAs as novel targets for therapy in hepatocellular carcinoma. *Pharmacol. Ther.* 161 67–78. 27013343 10.1016/j.pharmthera.2016.03.004 PMC4851900

[B27] Ponting C. P. Oliver P. L. Reik W. (2009). Evolution and functions of long noncoding RNAs. *Cell* 136 629–641. 19239885 10.1016/j.cell.2009.02.006

[B28] Portoso M. Ragazzini R. Brencic Z. Moiani A. Michaud A. Vassilev I. (2017). PRC2 is dispensable for HOTAIR-mediated transcriptional repression. *EMBO J.* 36 981–994. 28167697 10.15252/embj.201695335 PMC5391141

[B29] Quinodoz S. Guttman M. (2014). Long noncoding RNAs: an emerging link between gene regulation and nuclear organization. *Trends Cell Biol.* 24 651–663. 25441720 10.1016/j.tcb.2014.08.009 PMC4254690

[B30] Schmitt A. M. Chang H. Y. (2016). Long Noncoding RNAs in Cancer Pathways. *Cancer Cell* 29 452–463. 27070700 10.1016/j.ccell.2016.03.010 PMC4831138

[B31] Shan Y. Ma J. Pan Y. Hu J. Liu B. Jia L. (2018). LncRNA SNHG7 sponges miR-216b to promote proliferation and liver metastasis of colorectal cancer through upregulating GALNT1. *Cell Death Dis.* 9:722. 10.1038/s41419-018-0759-7 PMC6006356 29915311

[B32] Sun Y. Wei G. Luo H. Wu W. Skogerbo G. Luo J. (2017). The long noncoding RNA SNHG1 promotes tumor growth through regulating transcription of both local and distal genes. *Oncogene* 36 6774–6783. 28825722 10.1038/onc.2017.286

[B33] Thomson D. W. Dinger M. E. (2016). Endogenous microRNA sponges: evidence and controversy. *Nat. Rev. Genet.* 17 272–283. 27040487 10.1038/nrg.2016.20

[B34] Tsai M. C. Manor O. Wan Y. Mosammaparast N. Wang J. K. Lan F. (2010). Long noncoding RNA as modular scaffold of histone modification complexes. *Science* 329 689–693. 20616235 10.1126/science.1192002 PMC2967777

[B35] Vigouroux A. Oldewurtel E. Cui L. Bikard D. van Teeffelen S. (2018). Tuning dCas9’s ability to block transcription enables robust, noiseless knockdown of bacterial genes. *Mol. Syst. Biol.* 14:e7899. 10.15252/msb.20177899 PMC5842579 29519933

[B36] Wu C. Zhu X. Tao K. Liu W. Ruan T. Wan W. (2018). MALAT1 promotes the colorectal cancer malignancy by increasing DCP1A expression and miR203 downregulation. *Mol. Carcinog.* 57 1421–1431. 29964337 10.1002/mc.22868

[B37] Zhang P. F. Wang F. Wu J. Wu Y. Huang W. Liu D. (2018). LncRNA SNHG3 induces EMT and sorafenib resistance by modulating the miR-128/CD151 pathway in hepatocellular carcinoma. *J. Cell Physiol.* 234 2788–2794. 30132868 10.1002/jcp.27095

[B38] Zhang T. Cao C. Wu D. Liu L. (2016). SNHG3 correlates with malignant status and poor prognosis in hepatocellular carcinoma. *Tumour Biol.* 37 2379–2385. 26373735 10.1007/s13277-015-4052-4

